# Inhibition of broomrape germination by 2,4‐diacetylphloroglucinol produced by environmental *Pseudomonas*

**DOI:** 10.1111/1751-7915.14336

**Published:** 2023-10-27

**Authors:** Tristan Lurthy, Ségolène Perot, Florence Gerin‐Eveillard, Marjolaine Rey, Florence Wisniewski‐Dyé, Jordan Vacheron, Claire Prigent‐Combaret

**Affiliations:** ^1^ Ecologie Microbienne Université Claude Bernard Lyon1, Université de Lyon, CNRS UMR‐5557, INRAe UMR‐1418, VetAgro Sup Villeurbanne France; ^2^ Department of Fundamental Microbiology University of Lausanne Lausanne Switzerland

## Abstract

Parasitic weeds such as broomrapes (*Phelipanche ramosa* and *Orobanche cumana*) cause severe damage to crops and their development must be controlled. Given that phloroglucinol compounds (PGCs) produced by environmental *Pseudomonas* could be toxic towards certain plants, we assessed the potential herbicidal effect of the bacterial model *Pseudomonas ogarae* F113, a PGCs‐producing bacterium, on parasitic weed. By combining the use of a mutagenesis approach and of pure PGCs, we evaluated the in vitro effect of PGC‐produced by *P. ogarae* F113 on broomrape germination and assessed the protective activity of a PGC‐producing bacteria on oilseed rape (*Brassica napus*) against *P. ramosa* in non‐sterile soils*.* We showed that the inhibition of the germination depends on the PGCs molecular structure and their concentrations as well as the broomrape species and pathovars. This inhibition caused by the PGCs is irreversible, causing a brown coloration of the broomrape seeds. The inoculation of PGCs‐producing bacteria limited the broomrape infection of *P. ramosa*, without affecting the host growth. Moreover, elemental profiling analysis of oilseed rape revealed that neither F113 nor applied PGCs affected the nutrition capacity of the oilseed rape host. Our study expands the knowledge on plant‐beneficial *Pseudomonas* as weed biocontrol agents and opens new avenues for the development of natural bioherbicides to enhance crop yield.

## Graphical abstract

The present work expands our knowledge about the role that DAPG‐producing *Pseudomonas* play as natural bioherbicides to ward off parasitic plant infections, which are responsible for important yield losses in crops.

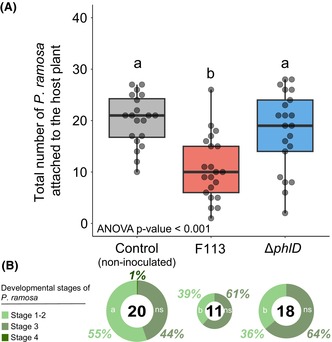

## INTRODUCTION

Broomrapes are parasitic plants causing significant damage to different crops in agroecosystems (Parker, 2012). They belong to the *Orobanche* and *Phelipanche* genera from the *Oroabanchaceae* family (Bennett & Mathews, 2006; Joel, 2009). These parasitic plants are obligate root holoparasites, entirely dependent on their host plant to survive as they are not capable of photosynthesis (Westwood, 2013). Indeed, these parasitic plants obtain all the resources they need while maintaining their host alive for accomplishing their entire life cycle (Cartry et al., 2021; Mutuku et al., 2021). The seed germination and haustorium formation are specifically induced by allelochemical signals such as strigolactones released from the host roots (Aliche et al., 2020). The activity spectrum of these parasitic plants can be either specific (e.g. *Orobanche cumana*, which can only parasitize sunflower) or generalist (e.g. *Phelipanche ramosa* able to parasitize tomato (*Solanum lycopersicum*), potato (*Solanum tuberosum*), hemp (*Cannabis sativa*), tobacco (*Nicotiana tabacum*) and oilseed rape (*Brassica napus*)) (Cartry et al., 2021; Parker, 2012). Broomrapes produce a large number of small seeds (less than 3 mm) that can survive in soil for several decades. This constitute the main problem to constrain their deleterious impact on crops (Haring & Flessner, 2018). The survival of the seeds depends on various abiotic factors (pH, humidity, climate) (Rubiales et al., 2003) and biotic factors (host plants, soil and rhizosphere microbiota; (Huet et al., 2020; Kawa et al., 2022; Martinez et al., 2023; Mutuku et al., 2021). Different agricultural strategies attempt to regulate broomrape populations in agroecosystems, such as crop rotation, triggering the suicidal germination of the plant parasitic seeds or the use of resistant host plant varieties or chemical herbicides (Cartry et al., 2021). However, biological control solutions are emerging to limit broomrape infestation, including the use of microorganisms (Cartry et al., 2021; Masteling et al., 2019). Indeed, several microorganisms inhibit the germination of different broomrape species, including, among others, *Fusarium oxysporum* (Hasannejad et al., 2006), *Azospirillum brasilense* (Dadon et al., 2004) or *Pseudomonas fluorescens* (Balthazar et al., 2021). Although the use of microorganisms represents a promising alternative to ward off parasitic plants, their mode of action as well as the identification of the metabolites responsible for their inhibition effect remains often uncharted.

Several environmental *Pseudomonas* are well known as plant‐colonizing bacteria (Silby et al., 2011). These *Pseudomonas* usually display a large arsenal of secondary metabolites encoded within their genomes that act on plant health and development (Haas & Défago, 2005; Loper et al., 2012). Among these metabolites, 2,4‐diacetylphloroglucinol (DAPG) has been studied notably for its role in plant protection. DAPG and its biosynthetic intermediates, phloroglucinol (PG) and monoacetylphloroglucinol (MAPG) are the main phloroglucinol compounds (PGCs) produced by *Pseudomonas* belonging to the *P. protegens* and *P*. *corrugata* subgroups (Almario et al., 2017). The production of DAPG relies on the presence of the *phl* gene cluster composed out of nine genes (Achkar et al., 2005; Biessy & Filion, 2021). The initiation of the synthesis of PG from malonyl‐CoA is mediated by *phlD* encoding a polyketide synthase, while *phlABC* encode for enzymes implicated into the transformation of PG to MAPG and subsequently to DAPG. The transformation of MAPG to DAPG is reversible through a hydrolase encoded by *phlG*. The remaining genes, *phlF/phlH* and *phlE*, are involved in the regulation as well as the secretion of these PGCs, respectively. The production of PGCs by *Pseudomonas* is influenced by environmental factors including (i) carbon sources (Shanahan et al., 1992), (ii) specific metabolites found in the root exudates such as flavonoids, apigenin and phloretin (Yu et al., 2020) or (iii) iron availability and interaction with other microorganisms (Laveilhé et al., 2022).

In addition to having been studied for its role in plant pathogen suppression, DAPG acts as a signal molecule affecting gene expression of plant‐beneficial traits in other microorganisms. Indeed, DAPG was described as an inducer of the production of PGCs and a repressor of the production of pyoluteorin in other *Pseudomonas* (Brodhagen et al., 2004; Maurhofer et al., 2004). Moreover, DAPG produced by *Pseudomonas* also activates the expression of genes involved in the production of auxins by *Azospirillum baldaniorum* Sp245, another plant‐beneficial microorganism (Combes‐Meynet et al., 2011). Since PGC‐producing *Pseudomonas* are residing in the vicinity of or on plant roots, PGCs produced diffuse and also interact directly with plant root cells. Thus, it was shown that DAPG elicited the plant‐induced systemic resistance (ISR), protecting partially the plant leaves from the oomycete *Peronospora parasitica* (Bakker et al., 2007; Iavicoli et al., 2003). On the root part, the addition of DAPG triggered a massive increase in the efflux of amino acids by plant root cells (Phillips et al., 2004). It was also demonstrated that DAPG modulates auxin‐dependent plant signalling pathway leading to significant modifications of plant root development (Brazelton et al., 2008; Vacheron et al., 2018; Weller et al., 2012). Moreover, following an exposition to DAPG, the germination as well as the development of different crop plants could be impacted (Brazelton et al., 2008; Keel, 1992; Khan et al., 2022). Nevertheless, this herbicidal effect remains variable according to the plant species and was observed following the exposure to high concentrations that do not reflect those produced in vivo.

In this study, we aimed to investigate the impact of PGCs on the germination of the two main parasitic plants, *Phelipanche* and *Orobanche*. To evaluate the herbicidal effects of these PGCs, we have investigated the inhibitory effect of PGCs‐producing strains and pure molecules in different in vitro and *in planta* experimental systems. First, the impact of the PGCs‐producing strain *Pseudomonas ogarae* F113 and its mutants, a PGC‐deficient and PGC overproducers, have been studied on broomrape germination by applying culture supernatants. Then, the role of PGCs on the germination of four different broomrapes was assessed at different concentrations under in vitro experiment. Finally, we evaluated the ability of PGCs‐producing bacteria and DAPG application to protect oilseed rape against broomrape in greenhouse, in non‐sterile soil.

## EXPERIMENTAL PROCEDURES

### Bacterial strains and media

We used the plant‐beneficial model strain *Pseudomonas ogarae* F113 (Garrido‐Sanz et al., 2021; Shanahan et al., 1992) and several of its mutants (Vacheron et al., 2018). The bacterial strains used in this study as well as their characteristics are listed in Table S1. The different bacterial strains were incubated at 28°C in King's B (King et al., 1954) medium or in a modified AB medium (ABm) supplemented with gentamycin (15 μg/mL when necessary) to maintain plasmid pBBR1‐MCS5‐*phlD*. ABm was composed of salts [MgSO_4_ (1.2 mM), CaCl_2_ (70 μM), NH_4_Cl (18 mM), KCl (2 mM), FeSO_4_ (9 μM)], a phosphate buffer diluted 10‐fold containing K_2_HPO_4_ (1.725 mM) and NaH_2_PO_4_ (960 μM) and fructose (20 mM) as carbon source.

### Plant material

Seeds of *Phelipanche ramosa* were collected in France as described in Huet et al., 2020 on winter oilseed rape (*Phelipanche ramosa* pv. oilseed rape), tobacco (*Phelipanche ramosa* pv. tobacco) and hemp (*Phelipanche ramosa* pv. hemp). Seeds of *Orobanche cumana* that parasites sunflower were provided by Terres Inovia in 2016 (Huet et al., 2020). Seeds of *Brassica napus* cultivar AMAZZONITE (broomrape‐sensitive) were provided by the breeder companies RAGT 2n (France).

### Chemicals

The germination of broomrape seeds was triggered using the synthetic strigolactone analogue GR24 (Chiralix, Nijmegen, NL). It was first suspended in acetone (4.79 mg/mL), then diluted at 10 μM with a phosphate buffer (1 mM sodium‐potassium phosphate buffer at pH 7.5).

Phloroglucinol (PG, Sigma‐Aldrich), mono‐acetyl‐phloroglucinol (MAPG, Cayman Chemical), 2,4‐diacetylphloroglucinol (DAPG, ChemCruz) and tri‐acetyl‐phloroglucinol (TAPG, Santa Cruz Biotechnology) were suspended in methanol (20 mM). These solutions were diluted with 1 mM phosphate buffer to obtain different stock solutions at different concentrations (66.60, 33.30, 16.65, 8.33, 4.16 μM). As methanol might have an effect on the germination of broomrapes, the final concentration was adjusted to 0.33% in all these stock solutions to prevent an effect of the dilution.

### Quantification of phloroglucinol compounds produced in bacterial supernatants

The quantification of PGCs was conducted on 1.5 mL of bacterial culture supernatants for each condition. First, supernatants were lyophilized (Martin Christ Alpha 1–4 LSC, Osterode, Germany) prior solid/liquid extraction with methanol. Samples were sonicated 20 min, then centrifuged for 20 min at 15,000 *g* and the supernatants recovered. The extraction protocol was repeated, leading to a total extracted volume of 3 mL per sample. The organic phase (methanol) was dried using a SpeedVac (Centrivap Cold Trap Concentrator; LABCONCO Co., MO, USA). Dried extracts were suspended in 200 μL of methanol and centrifuged for 5 min at 12,000 *g* to pellet the remaining solid phase.

Two hundred microlitres of the supernatant were then transferred into vials and were proceeded for ultra‐high pressure liquid chromatography coupled with UV (UHPLC‐UV) analysis, as previous described. (Rieusset et al., 2020). Chromatograms were analysed with MassHunter Qualitative Analysis B.07.00 software (Agilent Technologies) and the quantification of DAPG and other PGCs was done according to a standard curve with commercial PGCs.

### Inhibition of broomrape germination in vitro

Broomrape seeds were surface‐disinfected with minor modifications to the method as previously described (Pouvreau et al., 2013). Briefly, broomrape seeds were soaked 5 min in a bleach solution (9.6% active chlorine) and then washed 5 times with sterile water. After washing, 1 mM phosphate buffer supplemented with plant agar 0.1% and PPM 0.2% (Plant Preservative Mixture; Plant Cell Technology) was added to obtain a density of approximatively 2000 seeds/mL. These solutions containing the seeds were conditioned in sealed tubes for 10 days at 21°C in the dark in a cooled incubator (LMS, model 120, Kent, UK). The supernatant of conditioned seeds was removed and replaced by fresh phosphate buffer supplemented with plant agar 0.1% and PPM 0.2%. Fifteen microlitres of this seed suspension were distributed in a 96‐well plate (Cellstar; Greiner Bio‐One, France), corresponding to approximatively 30 seeds per well. Then, 10 μL of GR24 solution were then added in each well (final concentration of 1 μM). Seventy‐five microlitres of either bacterial supernatants (3‐fold diluted), or cocktails or individual PGCs were added to obtain a final volume of 100 μL per well. For the addition of PGCs, the 75 μL were taken from the different stock solutions described above to obtain final concentrations of 50, 25, 12.5, 6.25 and 3.125 μM. Negative controls were realized using 75 μL of fresh ABm medium fructose 20 mM for supernatant (3‐fold diluted) or phosphate buffer with 0.33% of methanol. After 10 days of incubation at 21°C in the dark, the percentage of broomrape germinated seeds was counted under a binocular (Leica, Switzerland) using the software Zen 2.3.

### Greenhouse experiments

*Brassica napus* plants were grown on a soil mix containing 1/3 of a natural loamy soil collected at the experimental farm in La Côte‐St‐André (France; 16.2% clay, 43.9% silt and 39.9% sand, pH 7.0, in water; 2.1% organic matter (El Zemrany et al., 2006)), 1/3 of vermiculite and 1/3 of TS3 peat‐based substrate (Klasmann‐Deilmann GmbH, Geeste, Germany). The humidity of the soil mix was maintained at 70% of field capacity. Each pot was filled with 1 L of free‐broomrape soil mix and then further filled with another litre of soil mix contaminated with non‐disinfected seeds of *Phelipanche ramosa* pv. oilseed rape leading to a final density of 3.9 mg of seeds per pot corresponding to approximatively 300 seeds per litre of soil. Seeds of *Brassica napus* cultivar AMAZZONITE were sown in pot after being pre‐germinated 24 h in the dark at 21°C in Petri dish containing water‐soaked Whatman paper.

The protective effect of the inoculation of F113 was compared to its mutant impaired in the production of PGCs (Δ*phlD*). The different bacterial strains were cultured 24 h in King's B medium at 28°C. The bacteria were centrifuged at 4500 rpm during 10 min and washed with a MgSO_4_ 10 mM solution before being adjusted to a bacterial concentration of 2 × 10^6^ CFU/mL. Five millilitres of these bacterial suspensions were sprayed at the base of the plant stem. Five millilitres of a MgSO_4_ 10 mM solution were applied as non‐inoculated control. In parallel, the impact of DAPG application was also assessed (more details in supporting information, Figures S5 and S6).

Twenty pots per conditions were used for the inoculation of bacteria. The experiment was conducted under controlled conditions with a 16 h light and 8 h dark photoperiod, at 25°C with 50%–70% relative humidity in a greenhouse for 50 days. Treatments were applied twice during the experiment. The first bacterial application was performed when *B. napus* had between 2 to 4 leaves. The second application was done at 6 to 8 leaves. After 50 days, the root systems were sampled and the adhering soil was very thoroughly removed by hands. Broomrapes attached to each root system were all collected, classified according to their development stage (using the infectivity scale described in Figure S4) and counted. The shoot and root dry biomasses of *B. napus* were also measured at the end of the experiment after 3 days at 70°C in an oven.

### Elemental profiling analysis of *B. napus*

After 50 days, dry samples from shoot of rapeseed from greenhouse experiment were ground into fine powder using a metal ball in a 50 mL plastic tube. For the elemental profiling analysis, 5 independent samples of 4 plants for strains‐inoculation and of 2 plants for DAPG treatment were collected. The concentrations of 17 elements (Na, Mo, Cd, Sb, Be, B, Mg, P, S, Ca, Mn, Fe, Co, Ni, Cu, Zn and K) were measured by High Resolution Inductively Coupled Plasma Mass Spectrometry (HR ICP‐MS, Thermo Scientific, Element 2TM, Bremen, Germany) as previously described (Lurthy et al., 2020).

### Data processing and statistical analysis

Data were analysed using R studio (v.4.2.1) and considered significantly different when *p*‐value <0.05. The data were assessed for Normal distribution and variance homogeneity using Shapiro–Wilk tests and Bartlett tests, respectively. When these parameters were respected, we performed ANOVA coupled with either HSD‐Tukey test (more than 3 conditions to compare) or LSD‐Fisher test (3 conditions to compare). Otherwise, Kruskal‐Wallis tests applying Bonferroni correction were used to detect differences between conditions. A two‐way ANOVA was performed to assess the effect of the different variable tested, such as the effect of the pathovar and / or the PGCs applied on broomrape seed germination.

## RESULTS

### The DAPG produced by *Pseudomonas* contributes to the inhibition of the germination of *Phelipanche ramosa* in vitro

We tested the ability of *Pseudomonas ogarae* F113, a PGC‐producing *Pseudomonas*, to inhibit the germination of four different broomrapes selected according to their host specificity, their parasitism cycle and their associated microbiota (Huet et al., 2020): *P. ramosa* pv. oilseed rape, *O. cumana* sunflower, *P. ramosa* pv. tobacco and *P. ramosa* pv. hemp.

**FIGURE 1:**
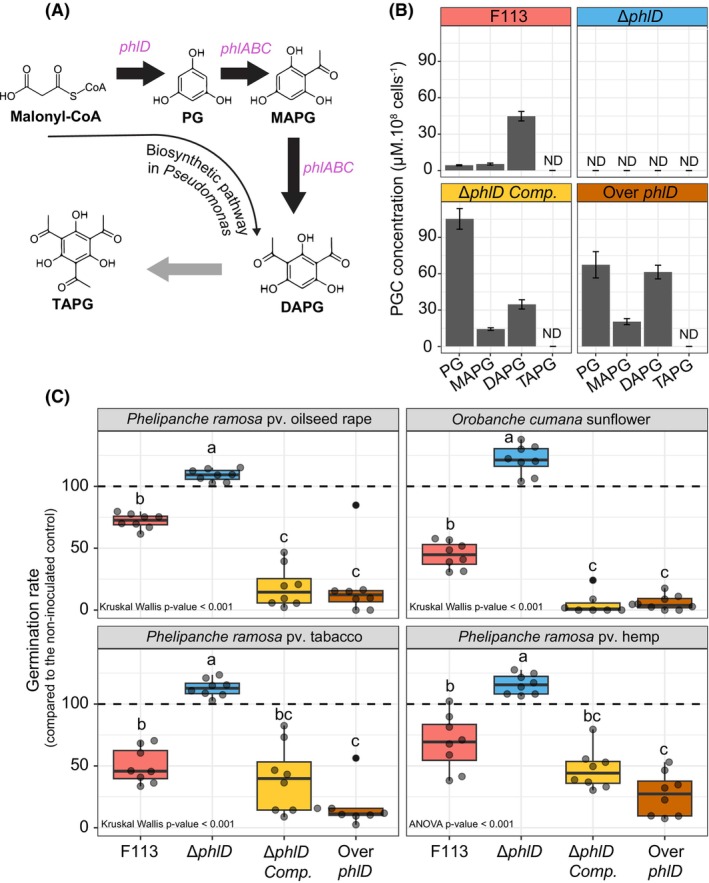
Impact of bacterial supernatants of PGC‐producing *Pseudomonas* on the germination rate of different broomrapes. (A) Biosynthetic pathway of the DAPG in *Pseudomonas* cells (according to Biessy & Filion, 2021). The genes involved in the different transformation steps are written in italic. (B) Quantification of Phloroglucinol (PG), monoacetylphloroglucinol (MAPG), diacetylphloroglucinol (DAPG) and triacetylphloroglucinol (TAPG) in the bacterial supernatants of *Pseudomonas ogarae* F113 and its mutant derivatives: Δ*phlD* (mutant impaired in the production of DAPG); Δ*phlD* Comp. (complemented mutant strain overproducing PG) and Over *phlD* (DAPG, MAPG and PG overproducing strain). Error bars correspond to the standard deviation; ND: Not detected. (C) Impact of these supernatants on the germination capacity of different *P. ramosa* pathovars and *O. cumana* in vitro. The supernatants as well as the control condition were supplemented with 1 μM of the germination stimulant (GR24). Results are expressed as percentage of germination of the non‐inoculated ABm medium control. Statistical differences were assessed by ANOVA and Kruskal‐Wallis test using a Bonferroni correction and are indicated with letters. The horizontal lines indicate the interquartile range with the centre representing the median.

We first determined the concentration of four different PGCs (PG, MAPG, DAPG and TAPG, Figure 1C) by UHPLC‐UV in the supernatant of *P. ogarae* F113 wild type (F113) as well as in the supernatants of a mutant impaired in the production of DAPG (Δ*phlD*), a complemented mutant (*ΔphlD* Comp.) and a strain engineered to overexpress the gene *phlD* (Over *phlD*) involved in the production of PG, the precursor of the DAPG (Figure 1A). Three out of the four different PGCs measured (PG, MAPG and DAPG) were detected in all supernatants, except in the supernatant of Δ*phlD* (Figure 1B). The concentration of DAPG in the supernatant of the complemented and the overproducing strains were in the same order of magnitude than F113. However, Δ*phlD* Comp. and Over *phlD* accumulated between 10 to 20 times more PG and MAPG in their supernatants than F113. Finally, TAPG was not detected in any of the bacterial supernatants (Figure 1B).

Then, we tested the capacity of the different bacterial supernatants to inhibit the germination of the different broomrapes (Figure 1C). We observed that the supernatant of F113 reduced the germination rate of all broomrapes tested (from 28% to 55%). However, the supernatant of Δ*phlD* did not inhibit the germination rate and appears, on the contrary, to slightly promote it compared to the control (>100%) (Figure 1C). The highest inhibition of the germination was observed with the supernatants of the complemented and the overproducing strains (Figure 1C). Furthermore, the sensitivity to the bacterial supernatants is different according to the broomrape tested (e.g. *P*. *ramosa* pv. tobacco being more inhibited by the supernatant of F113 than *P. ramosa* pv. oil seed rape) (Figure S1).

### Each PGC contributes differentially to the inhibition of broomrape germination

**FIGURE 2:**
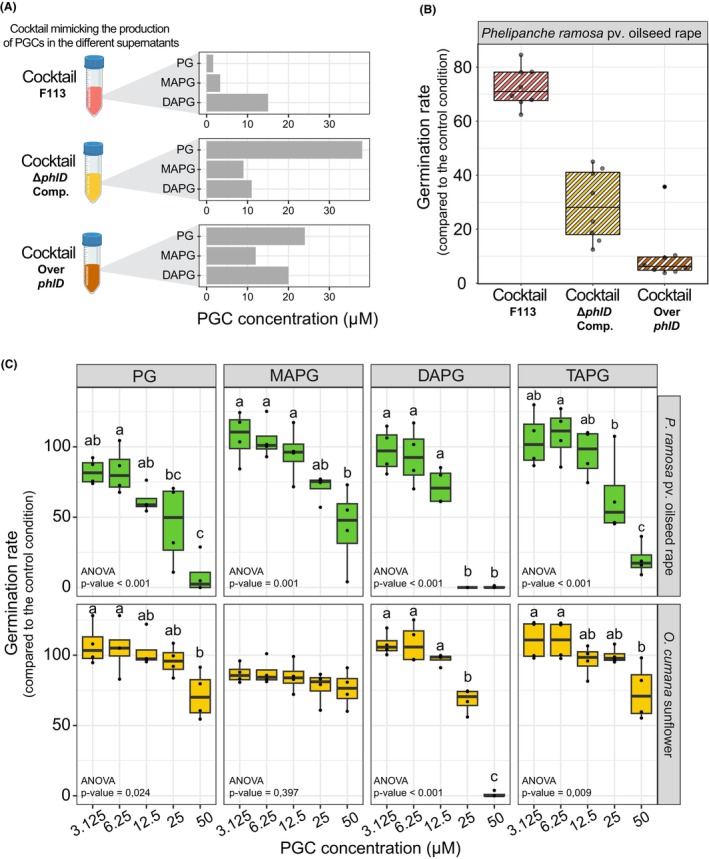
Individual contribution of the different PGCs to the inhibition of the germination of *P. ramosa* pv. oilseed rape and *O. cumana* sunflower. (A) Quantification of the pure PGCs added into the different cocktails mimicking the concentration detected in the bacterial supernatants. PG: Phloroglucinol; MAPG: monoacetylphloroglucinol; DAPG: Diacetylphloroglucinol; TAPG: Triacetylphloroglucinol. (B) Effect of the PGCs cocktails on the germination rate of *P. ramosa* pv oilseed rape. (C) Impact of PG, MAPG, DAPG and TAPG on the germination capacity of different *P. ramosa* pv. oilseed rape and *O. cumana* sunflower in vitro. In all the conditions, broomrape seed germination was induced by adding the germination stimulant GR24 (1 μM). Results are expressed as a percentage of germination of the non‐inoculated methanol control. Statistical differences were assessed by ANOVA and Kruskal‐Wallis test using a Bonferroni correction and are indicated with letters. The horizontal lines indicate the interquartile range with the centre representing the median. This experiment was repeated four times independently.

To assess the contribution of the PGCs detected in bacterial supernatants on broomrape germination, we composed three different cocktails made of commercially available PGCs, mimicking the proportions detected in the supernatants of the different F113 derivatives (Figure 2A). The application of these cocktails on seeds of *P. ramosa* pv. oilseed rape allowed us to determine whether the effect observed with the complex supernatants was mainly due to the presence of these PGCs and not to other compounds produced by the bacteria.

The same levels of inhibition were observed with the different molecular cocktails as in the experiment with bacterial supernatants. Indeed, the cocktails mimicking the concentration of PGCs in the supernatants of complemented and the overproducing strains most inhibited the germination of *P. ramosa* pv. oilseed rape (Figure 2B). Thus, as observed with complex supernatants, the inhibition of *P. ramosa* germination is dependent on the composition of the PGCs and their relative concentrations (Figure 2A,B).

Furthermore, we wanted to determine the individual contribution of these PGCs to the inhibition of broomrape germination by testing five different concentrations of each PGC (Figure 2C). First, we performed this inhibition assays on the four previously used broomrape species. However, the presence of 0.33% of methanol in the PGC solutions inhibited seed germination of *P. ramosa* pv. tobacco and *P. ramosa* pv. hemp (Data not shown). Contrariwise to what was observed with the bacterial supernatants (Figure S1), the germination rate of *P. ramosa* pv. oilseed rape was more affected following the exposure to PGCs than *O. cumana* sunflower (two‐way ANOVA showed a broomrape effect, *p*‐value <0.001) (Figure 2C). Indeed, a reduction of more than 50% of *O. cumana* germination was observed only where the seeds were exposed to 50 μM of DAPG. Remarkably, MAPG did not affect the germination rate of *O. cumana*. On the contrary, the germination rate of *P. ramosa* pv. oilseed rape started to be affected by the addition of PGCs at 12.5 μM. DAPG was the most effective PGC to disable broomrape germination with 100% of inhibition at 25 μM and 50 μM for *P. ramosa* pv. oilseed rape and 100% inhibition at 50 μM for *O. cumana* sunflower. Indeed, the estimated median lethal concentration (LC50) is the lowest for the DAPG for both broomrape species tested (20.0 μM for *P. ramosa* pv. oilseed rape and 29.1 μM for *O. cumana* (Figure S2). At the end of the experiment, we recovered the seeds of *P. ramosa* treated with 50 μM of the different PGCs and washed those 3 times with phosphate buffer to remove PGC traces. Then we added again the GR24 germination stimulant. We observed that the inhibition by the PGC is irreversible since none of the seed treated with PGC germinated. Moreover, for all PGCs except for DAPG, between 25 and 50 μM, the inhibition effects were associated with a brown coloration of the seeds or the radicles (Figure S3).

### The inoculation of PGC‐producing *Pseudomonas* reduced the infection of *P. ramosa* on *Brassica napus*

**FIGURE 3:**
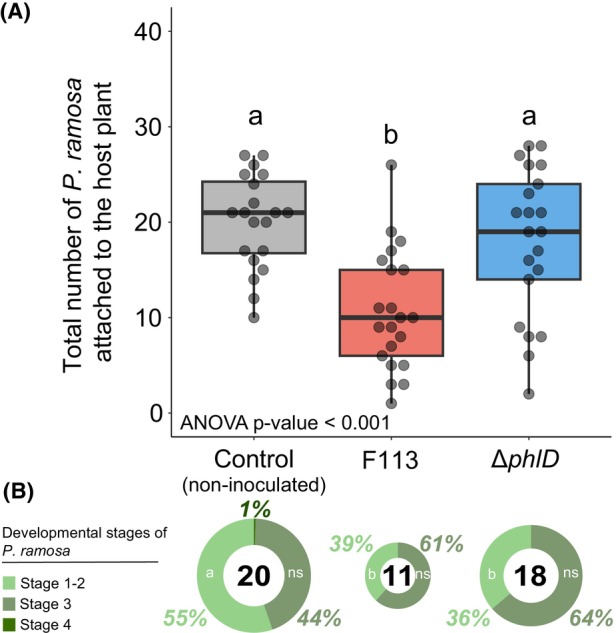
Impact of the inoculation of PGC‐producing *Pseudomonas* on the infection level by *P. ramosa* pv. oilseed rape on *Brassica napus* in greenhouse conditions. (A) Evaluation of the number of attached *P. ramosa* on the root system of *Brassica napus* after 50 days in the greenhouse. Different treatments were applied as follows: bacterial inoculants (F113 and Δ*phlD* impaired in the production of DAPG, approximately five millilitres per pot of solutions at 2 × 10^6^ bacteria/mL) and the control condition which corresponds to the application of 5 mL with MgSO_4_ 10 mM. This experiment was performed in a mixture containing natural soil artificially infested with approximatively 300 *P. ramosa* seeds per litre of soil. (B) Proportion of broomrapes attached to the root of *B. napus* according to their developmental stage. The developmental stage of *P. ramosa* was estimated according to the developmental scale available in Figure S4. The number in the centre of pie chart represents the mean of attached *P. ramosa* on *B. napus* roots, and the size of pie charts is proportional to this number. For A and B, Statistical differences are indicated with letters (ANOVA and Fisher's LSD tests, *p* < 0.05). The horizontal lines indicate the interquartile range with the centre representing the median.

**FIGURE 4:**
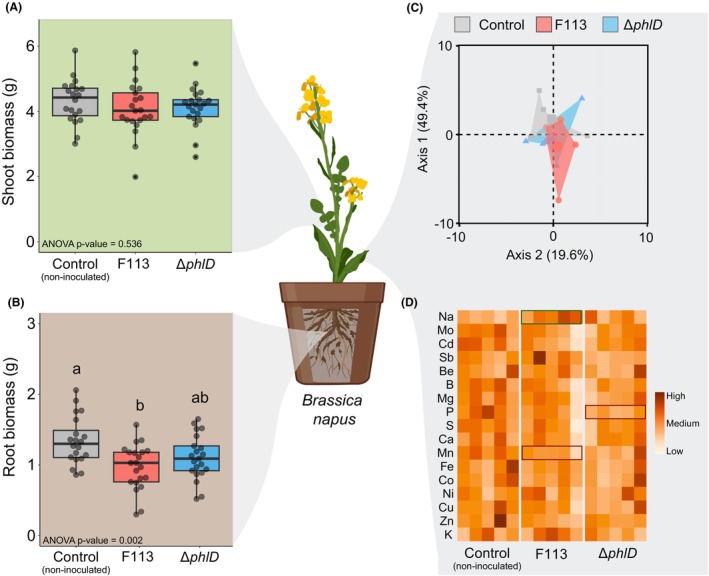
Effect of the inoculation of bacteria on the shoot (A) and root (B) biomasses and, on the ion profile (C,D) of the shoot of *Brassica napus*. (A,B) We measured for all the tested conditions (presented in Figure 3) the shoot and root biomasses of *Brassica napus*, 50 days after sowing. The control condition of the bacterial inoculant experiment corresponds to the application of 5 mL with MgSO_4_ 10 mM. The brown background of the boxplots corresponds to the root biomass data while the green background is associated with shoot biomass data. Statistical differences are indicated with letters (ANOVA and Fisher's LSD tests, *p* < 0.05). The horizontal lines indicate the interquartile range with the centre representing the median. (C) Principal component analysis of the element composition of the shoot of *Brassica napus* according to the different treatments applied. (D) Heatmap showing the elemental profile of the different conditions. Significant differences between treatments and control were obtained with Student's *t*‐test. When Student's *t*‐test assumptions were not met, a Welch *t*‐test was performed. Red and green boxes correspond to significant negative and positive impact on ionome, respectively. The detailed data are available in Table S2.

As the DAPG showed the best inhibition of broomrape germination in vitro, we compared the ability of F113 and its Δ*phlD* mutant to reduce the infection of *Brassica napus* by *P. ramosa* pv. oilseed rape in greenhouse conditions. We evaluated the developmental stage of *P. ramosa* using the development scale available in the Figure S4 and based on Martinez et al. (2023). We observed a significant reduction (47%) in the number of *P. ramosa* attached to the root of oilseed rape when F113 was inoculated (Figure 3A). This effect was not detected when the Δ*phlD* mutant was inoculated. Interestingly, at the end of the experiment, we observed a significant reduction in the proportion of early‐stage infections (stage 1 and 2; qualitative infection scale available in Figure S4) in the condition inoculated with F113 or Δ*phlD* compared to the control (Figure 3B). The proportion of broomrapes in a more advanced infection stage (i.e. particularly from stage 3, buds) did not change with bacterial inoculation. However, the DAPG‐producing F113 strain tended to decrease the average number of broomrapes in an advanced stage as it reduced significantly the total number of broomrapes per root system. We also measured the effect of the bacterial inoculation on the physiology of *Brassica napus* (Figure 4A–D **)**. We observed that the biomass of the root system was significantly lower than the control condition when F113 was inoculated (Figure 4B). We also looked at the nutrition capacity of *B. napus* using an elemental profiling approach (i.e. ionomic) to investigate potential switch of elements according to the treatments we applied. For all the tested conditions, we did not observe a main significant switch of the ion profile inside the shoots of *B. napus* (Figure 4C,D; Table S2). However, the proportions of certain ions changed according to the treatment applied. The inoculation of F113 led to a significant increase in sodium and a decrease in manganese quantity, whereas the mutant Δ*phlD* was associated with a decrease in potassium (P) (Figure 4C).

On the contrary, the application of DAPG at 50 or 250 μM (*v* = 5 mL) did not affect the infection of *P. ramosa* (Figure S5A,B), nor rapeseed biomasses (Figure S7A). Regarding elemental profiling analysis, the addition of DAPG did not switch as well and was associated with a significant increase of antimony (Sb), beryllium (Be) and a reduction of nickel (Ni) in rapeseed shoots (Figure S7B, C; Table S3).

## DISCUSSION

PGC‐producing *Pseudomonas* are well described for their capacity to protect different crop plants from the infection by plant pathogens. Their biocontrol activity has been shown to be dependent on the production of antimicrobial compounds, including DAPG. In this study, we assessed whether the biocontrol activity of such bacterial strains can be extended to the protection of crops towards parasitic plants. We focused on the model environmental *Pseudomonas*, *P. ogarae* F113, which is known to display different plant‐beneficial properties including the production of DAPG (Redondo‐Nieto et al., 2012; Vacheron et al., 2018).

Except for TAPG whose bacterial production remains to be demonstrated, F113 is able to synthesize, in different amounts, a cocktail of PGCs composed of PG, MAPG and DAPG (Figure 1B). It is worth noting that the production and the proportion of PGCs are variable among *Pseudomonas* strains (Duffy & Défago, 1999) and depend on many environmental factors such as carbon sources (Shanahan et al., 1992), bacterial lifestyle (e.g. planktonic or biofilm) (Rieusset et al., 2020) or composition of plant exudates (de Werra et al., 2011; Rieusset et al., 2021). The introduction of the *phlD* gene on a low‐copy plasmid in the *phlD* mutant (*ΔphlD* Comp.) and in the wild‐type F113 strain (Over *phlD*) modified the production of PGCs, these strains accumulating more PG and MAPG in their supernatants than F113. The production of different PGCs (as in the Over *phlD* F113 derivative) leads to a stronger inhibitory effect on the germination of the different broomrapes tested (Figure 1C). We also confirmed these results using a cocktail of commercial PGCs in the same proportions (Figure 2A,B) and highlighted that each of the PGCs displayed a different inhibition capacity towards the germination of broomrapes (Figure 2C). Indeed, we showed that the DAPG provided the highest inhibition results. Islam and von Tiedemann (2011) tested the effect of DAPG and its derivatives on the zoosporogenesis and the motility of zoospores from *Plasmopara viticola* and *Aphanomyces cochlioides*. They also observed that DAPG displayed the highest inhibitory effect compared to its derivatives. Similar results were observed on the mycelial growth of the plant pathogen *Pythium ultimum* (de Souza et al., 2003). Altogether, these results highlight the importance to consider the amount of the different precursors of DAPG produced by PGCs‐producing *Pseudomonas* strains as they could also act as active compounds.

Although DAPG had been well described for its antimicrobial activity towards different kind of plant pathogens (e.g. fungi, oomycetes) (Biessy & Filion, 2021), its toxic effect on plants was also investigated on crop plants and appeared to be associated with its concentration and the plant species (Keel, 1992). A recent study analysed the impact of DAPG at 50 μg mL^−1^ (equivalent to 240 μM) on the germination rate of 69 wheat cultivars and showed strong differences according to cultivars (Yang et al., 2021). This is in line with our results where the inhibition of germination was dependent on the concentration of DAPG and also different according to the broomrapes species and pathovar. Chae et al. (2020) identified several genes implicated in the sensitivity of *Arabidopsis thaliana* to exposure to DAPG (Chae et al., 2020). These genes are involved in different metabolic pathways such as the tryptophan and monocarboxylic acid metabolisms and iron management (Chae et al., 2020). The development of molecular tools to modify broomrape genomes would provide new insights on the molecular determinants responsible for their resistance and sensitivity towards PGCs.

The mode of action of DAPG and its derivatives at the cellular level remains elusive. In our study, we observed a brown coloration of the broomrape radicles treated with PGCs (Figure S3). This coloration had previously been observed on tomato seedlings following the addition of 50 μM of DAPG (Brazelton et al., 2008). At cellular levels, the sensitivity to DAPG was correlated with disruption of F‐actin cytoskeleton in *A. cochlioides* hyphae (Islam & Fukushi, 2010) and with alterations of major physiological functions in yeast including the regulation of cellular responses to reactive oxygen stress and cell homeostasis (Kwak et al., 2011). Thus, the brown coloration of root tissues could be attributed to cell wall disorganization (Islam & Fukushi, 2010), impairment of mitochondrial functions (Kwak et al., 2011; Troppens et al., 2013) and induction of oxidative burst (Kwak et al., 2011). Furthermore, in our experiment, the damage caused by the DAPG on broomrape seeds was irreversible since none of the broomrape seeds treated with DAPG were able to germinate after several washing steps and the addition of the GR24 germination inductor.

To determine if PGCs‐producing *Pseudomonas* strains are good candidates to prevent the infection of broomrapes, we performed a greenhouse experiment with a natural soil, which was infected with broomrape seeds. The density of broomrape seeds used in this experiment was in the same range of population levels than those used in literature (Bernhard et al., 1998; Chen et al., 2016), leading to a number of attached broomrapes to *Brassica napus* root system close to what we observed in fields. We observed that the wild‐type F113 was able to significantly reduce the number of broomrapes bound to *B. napus* roots compared to the mutant impaired in DAPG production (Figure 3). Interestingly, the number of broomrapes associated with new host infection (stage 1 and 2) in the condition where the bacteria were inoculated was significantly lower than in the control condition. Thus, the reduction of the infection is delayed and did not start immediately after the inoculation of the bacterial strains. This lag phase could be interpreted as the time needed for the bacterial inoculant to establish itself within the soil and/or root microbiome, and/or to produce DAPG in sufficient amounts. We observed that the *ΔphlD* mutant was also able to reduce the proportion of broomrapes in an early infection stage. This could be due to the fact bacteria can exert, as well, an indirect positive activity on oilseed rape leading to a decreased sensitivity to broomrape infection, independently of the production of DAPG, as cited previously by Cartry et al. (2021). Moreover, several reports showed that DAPG act as a signalling molecule inducing the plant systemic resistance (Weller et al., 2012) or the expression of its own biosynthetic genes (Maurhofer et al., 2004). Thus, the enrichment of the *B. napus* rhizo‐microbiota with PGC‐producing pseudomonads could stimulate other PGC‐producing strains already present in the rhizosphere, leading to an increase of PGC concentrations in soil, thereby leading to detrimental effects on broomrape germination.

In addition, the inoculation of F113 leads to a decrease of the root system biomass of *B. napus* (Figure 4). It has been shown that the inoculation of PGC‐producing *Pseudomonas* was linked to a decrease of the root length in *Arabidopsis thaliana* and other crop plants (Brazelton et al., 2008; De Leij et al., 2002; Vacheron et al., 2018). Thus, PGCs‐producing strains could limit the germination of broomrapes by two modes of action. The first one can be direct via the production of DAPG that inhibit the parasite seed germination whereas the second affects the root length of the host plant that *in fine* decreases the probability of contact between the host plant roots and broomrape seeds within the rhizosphere soil.

Conversely, the application of DAPG did not limit the infection of *P. ramosa* on *B. napus* roots (Figure S5). The DAPG applied may have not only specifically targeted the broomrape seeds but also interacted with soil particles or the resident soil/root microbiomes as well as the oilseed plant. Thus, the amount of DAPG targeting the broomrape seeds might be insufficient to deliver a significant reduction of *P. ramosa* infection. Moreover, the soil chemical properties could impact the efficiency of DAPG inhibition. Indeed, Kwak and collaborators documented that DAPG has a half‐life of less than 6 h in soil (Kwak et al., 2012). Increasing the DAPG concentration over 250 μM might induce negative effects on the environment as any phytochemical products. Moreover, its field application would be too expensive. So, the application of pure DAPG has an herbicide against broomrape is neither realistic nor desirable. Conversely, we showed that a DAPG‐producing strain was able to significantly reduce broomrapes' infectivity under greenhouse conditions, suggesting that inoculation of PGC‐producing strains represents a better biocontrol solution against broomrapes. Except using genetically modified organisms, such as our strain Over *phl*D, which is banned in Europe, the application of PGC‐overproducing strains may improve considerably biocontrol effect.

Elemental analyses highlighted no unwanted changes on plant host nutrition induced by bacterial inoculant (Figure 4), or by pure DAPG amendment (Figure S7). It's an important concern in field where oilseed rape nutrition must be unchanged to achieve high yield. Shoot accumulation of sodium (*p*‐value <0.05 for F113 treatment and <0.1 for DAPG 50 μM treatment) appears to be linked to less parasitism. Sodium accumulation is generally considered as toxic for plants depending on plant species (Kronzucker et al., 2013). In our study, the accumulation of sodium does not affect the plant host but is correlated to a reduction of *P. ramosa* infection. Bacterial inoculation with *Pseudomonas* or *Azospirillum* was rather associated with a decrease of sodium in rapeseed leaf when exposed to harmful effects of salt stress (Farhangi‐Abriz et al., 2020). Thus, improvement of sodium uptake in the host by DAPG‐producing bacteria could be another factor associated with broomrape protection. As the nutrient flux from the host plant to the parasite is driven by an osmotic pressure differential between them (Shen et al., 2006), we hypothesize that the increase of sodium content in the host may reduce the level of nutrient uptake by the parasite and may impact its growth. Thus, sodium accumulation could be an interesting factor to study on oilseed rape cultivars in response to biocontrol agents. Nonetheless, the signals emitted by the broomrape and the *B. napus* roots during the haustoriogenesis that may attract biocontrol agents or participate to the regulation of their activities, are not yet precisely known, and their identification deserved particular attention.

Here, we showed that the F113 DAPG‐producing strain combined different direct and indirect effects to protect oilseed rape against broomrape. DAPG and its derivatives could be interesting bioherbicides produced by *Pseudomonas* for preventing parasitic plant infestation as we did not observe any toxicity towards the host plant. The soil and root microbiome compositions were previously associated with natural suppressiveness towards parasitic weeds. Indeed, several studies claimed that the suppressiveness of *Orobanche* sp. as well as *Striga hermonthica* is associated with the presence of specific bacterial taxa, including *Pseudomonas* (Kawa et al., 2022; Zermane et al., 2007). Since, PGC‐producing *Pseudomonas* can be followed in soil via qPCR approaches (Almario et al., 2013), determining the correlation between the community of PGC‐producing *Pseudomonas* and the level of parasitic plant infection would bring new insights on the ecological role of these bacteria in suppressive soils.

Finally, this study reinforces the interest of using microorganisms as natural solutions to regulate populations of pests and plant pathogens (Vurro, 2023). Indeed, this study is the first to discover and demonstrate the inhibitory effect of DAPG on parasitic plant germination, under in vitro conditions expanding the repertoire of plant‐beneficial properties of environmental pseudomonads. Furthermore, we evidenced that PGCs produced by *P. ogarae* are the main compounds that irreversibly inhibit the germination of broomrape species tested. Without the use of adjuvants able to enhance their biocontrol activity (Campos et al., 2023) and taking into account that PGC production could be modulated by *B. napus* and other microorganisms in the plant rhizosphere (Laveilhé et al., 2022), we obtained promising results during the experiments carried out in natural soil in greenhouse condition. Indeed, the inoculation of *P*. *ogarae* halved the infection of *P. ramosa* on its host plant, *Brassica napus*. The present work significantly expands our knowledge about the role that these plant‐beneficial *Pseudomonas* play in the environment. It provides new alternative direction for the development of natural bioherbicides to ward off parasitic plant infections, which are responsible for important yield losses in crops.

## AUTHOR CONTRIBUTIONS

**Tristan Lurthy:** Conceptualization (lead); data curation (lead); investigation (lead); methodology (lead); writing – original draft (lead); writing – review and editing (lead). **Ségolène Perot:** Investigation (equal). **Florence Gerin‐Eveillard:** Investigation (equal). **Marjolaine Rey:** Conceptualization (equal); investigation (equal). **Florence Wisniewski‐Dyé:** Conceptualization (supporting); investigation (supporting); writing – review and editing (lead). **Jordan Vacheron:** Conceptualization (lead); data curation (lead); investigation (lead); writing – review and editing (lead). **Claire Prigent‐Combaret:** Conceptualization (lead); data curation (lead); funding acquisition (lead); investigation (lead); project administration (lead); writing – review and editing (lead).

## CONFLICT OF INTEREST STATEMENT

The authors declare no conflict of interest.

## Supporting information


supporting information
