# Supplementary material for: Inhibition of broomrape germination by 2,4‐diacetylphloroglucinol produced by environmental Pseudomonas
Source: Microb Biotechnol. 2023 Oct 27;16(12):2313–25. doi: 10.1111/1751-7915.14336 (PMC10686154; doi:10.1111/1751-7915.14336)
Supplement: Supplementary file 1 — supporting information [file MBT2-16-2313-s001.docx]

**SUPPORTING INFORMATION**

**Inhibition of broomrape germination by 2,4-diacetylphloroglucinol produced by environmental *Pseudomonas.***

Tristan Lurthy^1#^, Ségolène Perot^1^, Florence Gerin-Eveillard^1^, Marjolaine Rey^1^, Florence Wisniewski-Dyé^1^, Jordan Vacheron^2#*^ & Claire Prigent-Combaret^1#*^

^1^ Ecologie Microbienne, Université Claude Bernard Lyon1, Université de Lyon, CNRS UMR-5557, INRAe UMR-1418, VetAgro Sup, 43 Boulevard du 11 Novembre 1918, 69622 Villeurbanne, France.

^2^ Department of Fundamental Microbiology, University of Lausanne, Lausanne, Switzerland.

*: co-senior authors

**Corresponding authors:** [tristan.lurthy@univ-lyon1.fr](mailto:tristan.lurthy@univ-lyon1.fr), [Jordan.Vacheron@unil.ch](mailto:Jordan.Vacheron@unil.ch), [claire.prigent-combaret@univ-lyon1.fr](mailto:claire.prigent-combaret@univ-lyon1.fr)

**Supporting Figures**

**Figure S1:** The sensitivity of broomrapes to bacterial supernatants is species and pathovar dependent.

**Figure S2:** Dose-response of two different broomrape species following their *in vitro* exposure to different PGCs.

**Figure S3:** The exposure of *P. ramosa* seeds to PGCs (PG, MAPG, DAPG or TAPG) and supernatants from PGCs-producing *Pseudomonas* strains leads to the apparition of a brown coloration.

**Figure S4:** Infectivity scale of *Phelipanche ramosa* during the greenhouse experiments.

**Figure S5:** Impact of pure DAPG on the infection level by *P. ramosa* pv. oilseed rape on *Brassica napus* in greenhouse conditions.

**Figure S6:** Evaluation of the impact of MeOH 1.5% contained in buffer on the infection level by *P. ramosa* pv. oilseed rape on *Brassica napus* in greenhouse conditions.

**Figure S7:** Effect of the application of DAPG on the root and shoot biomasses and, on the ion profile of the shoot of *Brassica napus*

**Supporting Tables**

**Table S1:** List of the bacterial strains used in this study.

**Table S2:** Impact of the inoculation of F113 or Δ*phlD* on the elemental composition of the leaves of *B. napus*. (µg/g of dry biomass, except for Mg, P, S, Ca, Fe and K i.e., mg/g of dry biomass).

**Table S3:**Impact of the application of two concentrations of DAPG on the elemental composition of the leaves of *B. napus*. (µg/g of dry biomass, except for Mg, P, S, Ca, Fe and K i.e., mg/g of dry biomass).


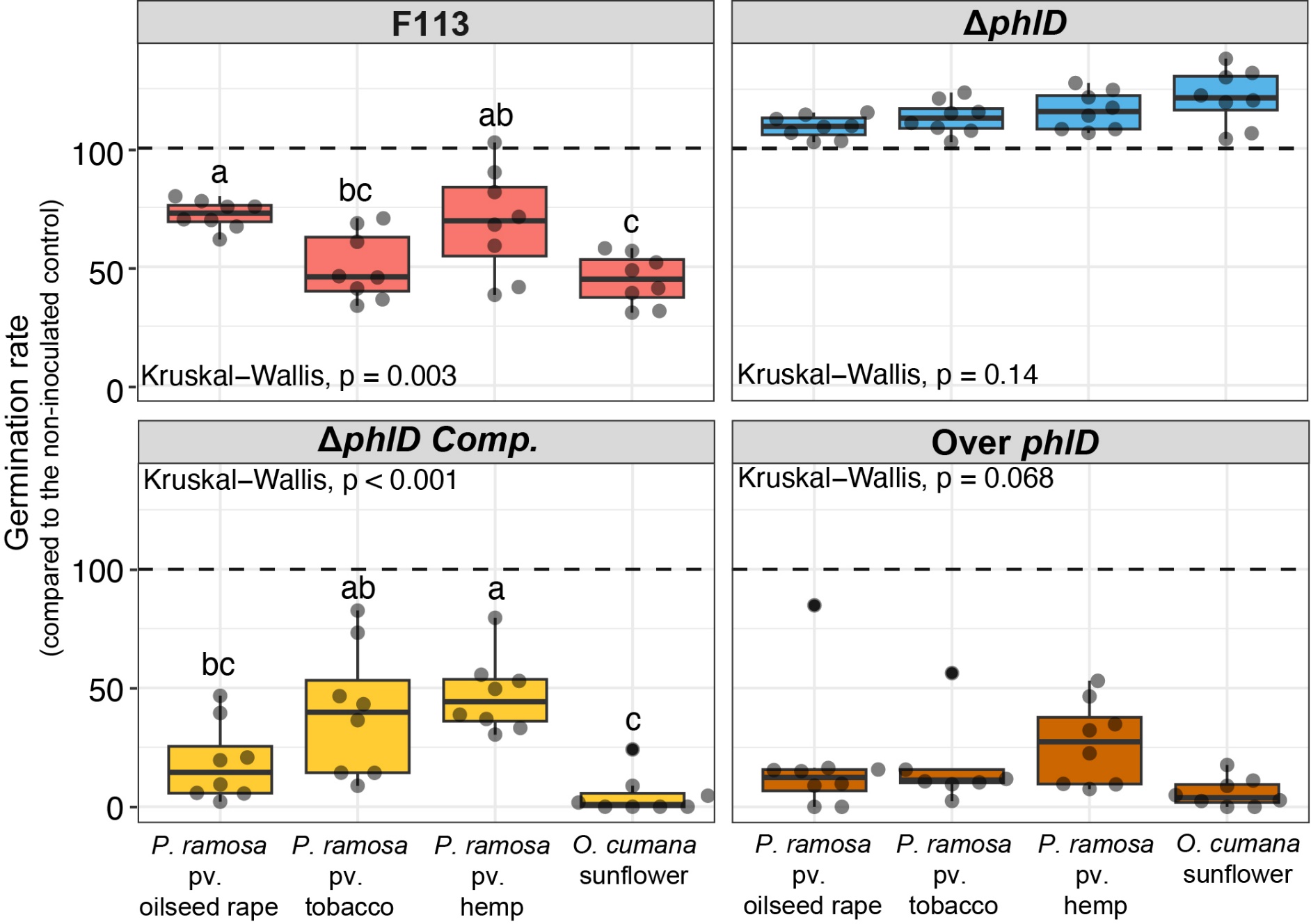


**Fig. S1:** **The sensitivity of broomrapes to bacterial supernatants is species and pathovar dependent.** Impact of supernatants from F113 and its derivatives on the germination capacity of different broomrapes *in vitro*. The supernatants as well as the control condition were supplemented with 1 µM of the germination stimulant (GR24). Results are expressed as percentage of germination of the non-inoculated control. Statistical differences were assessed by ANOVA and Kruskal-Wallis test using a Bonferroni correction and are indicated with letters. The horizontal lines indicate the interquartile range with the center representing the median.


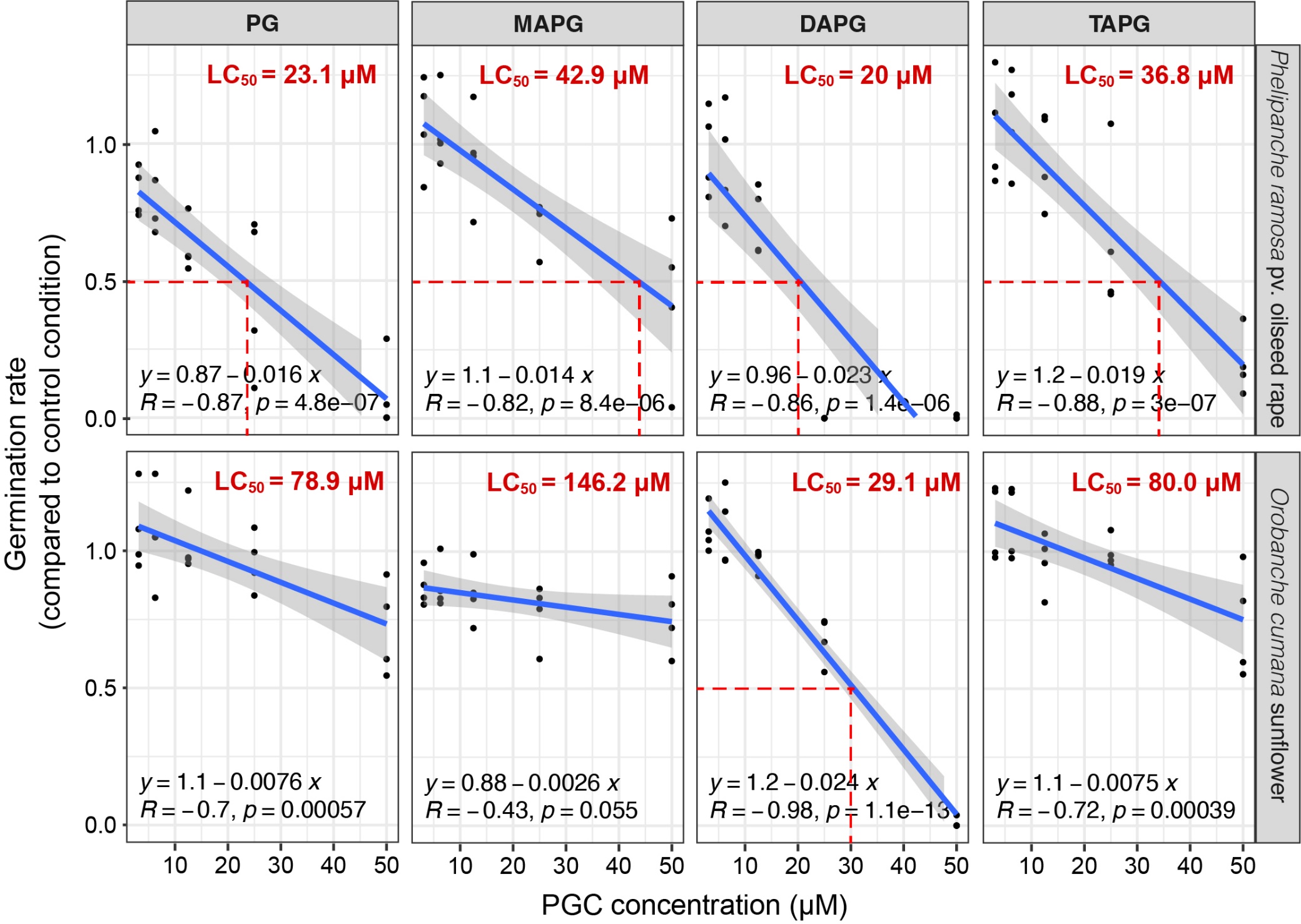


**Fig. S2:** **Dose-response of two different broomrape species following their *in vitro* exposure to different PGCs.** The LC50 (Lethal concentration 50) was estimated thanks to a linear regression model. Correlation coefficient were calculated using Spearman correlation test. PG: Phloroglucinol; MAPG: Monoacetylphloroglucinol; DAPG: Diacetylphloroglucinol; TAPG: Triacetylphloroglucinol.


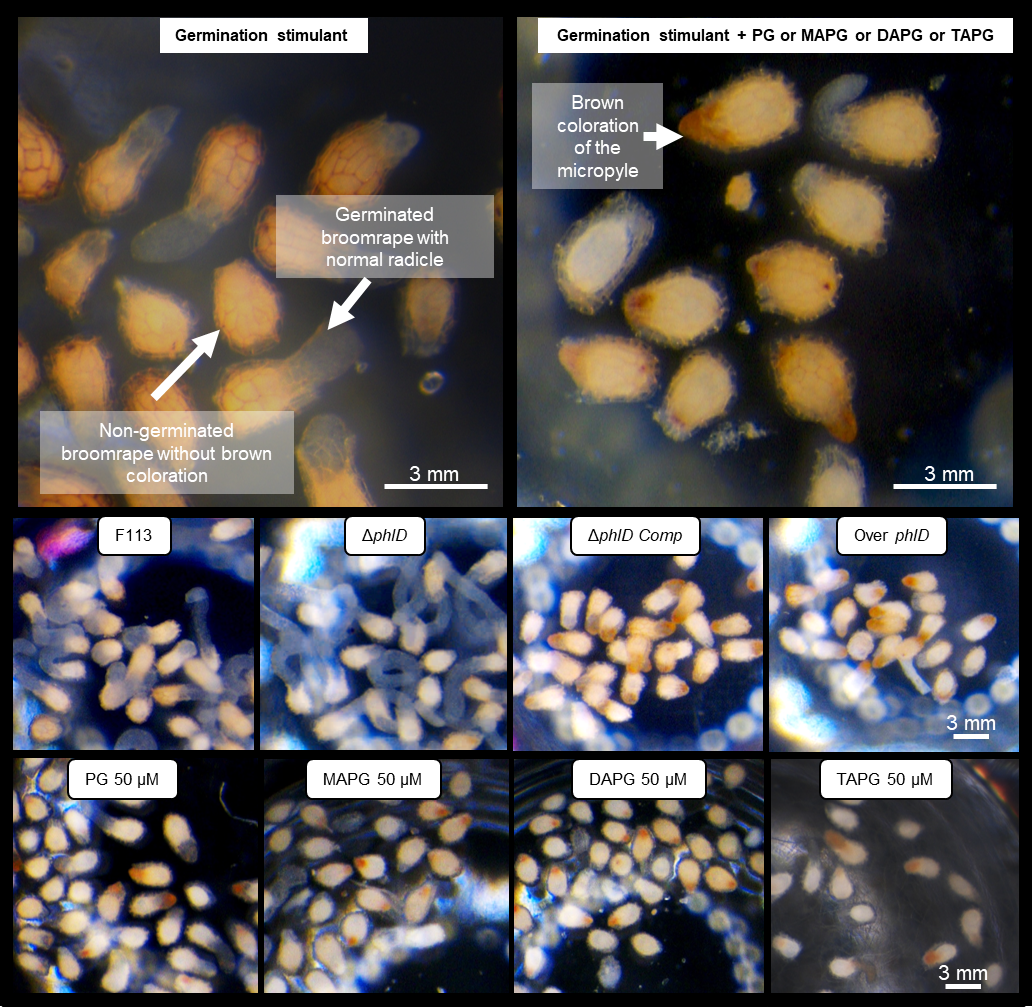


**Fig. S3:** **The exposure of *P. ramosa* seeds to PGCs (PG, MAPG, DAPG or TAPG) and supernatants from PGCs-producing *Pseudomonas* strains leads to the apparition of a brown coloration.** Seeds were photographed using camera (AxioCam MRc5) attached to binocular loupe, zoom x20.





**Fig. S4: Infectivity scale of *Phelipanche ramosa* used during the greenhouse experiments.** *Stage 1* corresponds to small tubercules without spikes (<2 mm of diameter); *Stage 2 Spider* corresponds to nodule with spikes without visible broomrape bud; *Stage 3* *Bud* corresponds to the apparition of broomrape bud; *Stage 4* *Stem* encompasses the development of floral stem and its emergence outside the soil.

**A**

**B**


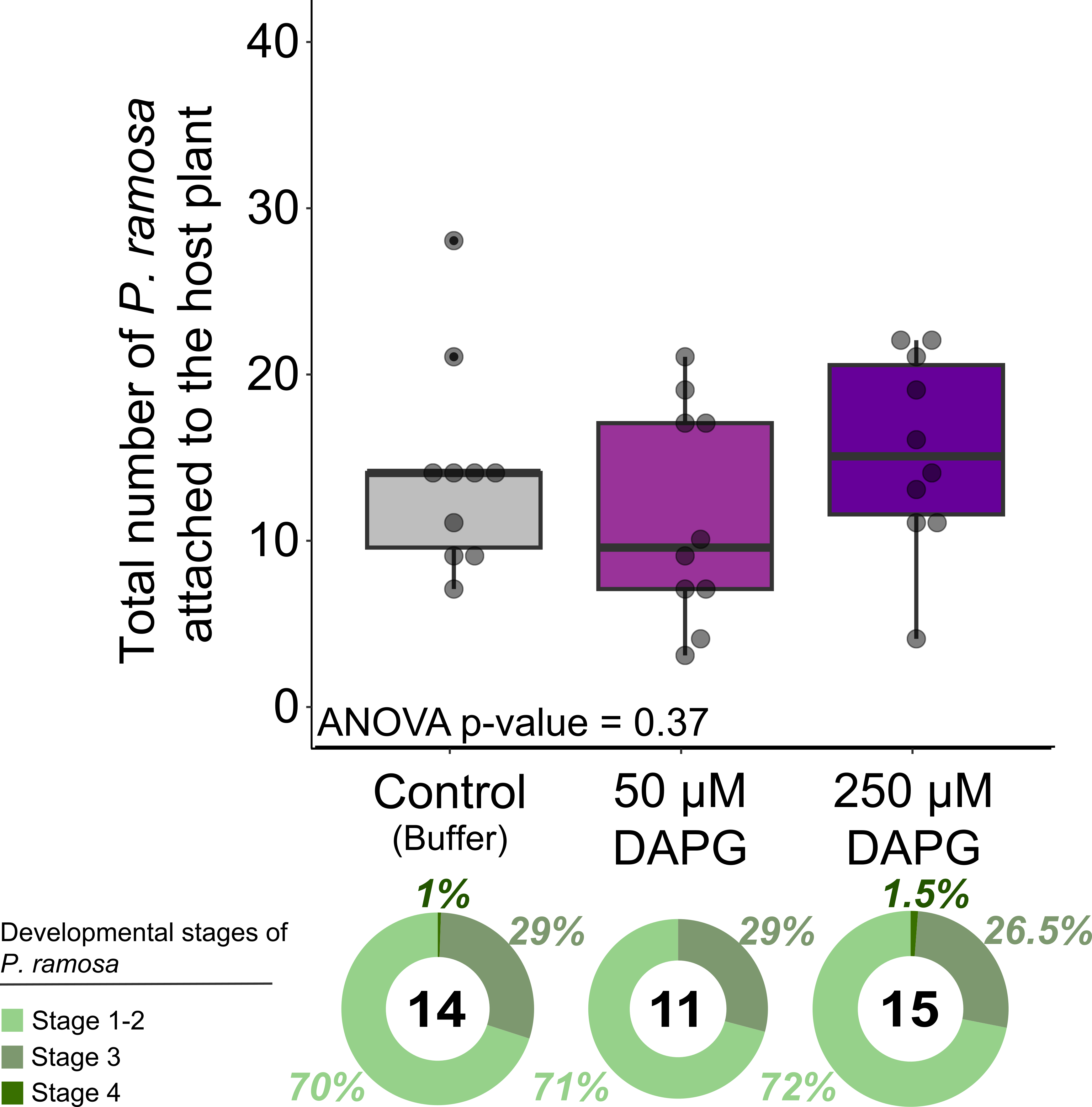


**Figure S5: Impact of pure DAPG on the infection level by *P. ramosa* pv. oilseed rape on *Brassica napus* in greenhouse conditions**. **(A)**: Evaluation of the effect of DAPG 50 µM or 250 µM on the number of attached *P. ramosa* on the root system of *Brassica napus* after 50 days in the greenhouse. Five milliliters of a solution concentrated at 50 µM or 250 µM were applied, respecting the same amount of MeOH solvent of 1.25% (e.g. 10 ml of solution DAPG 250 µM = 125 µl solution DAPG 20mM + 9.875 ml of 1 mM phosphate buffer pH 7.5). The control condition corresponds to the application of 5 ml with phosphate buffer supplemented with 1.25% of methanol. The impact of MeOH 1.25% on the number of attached *P. ramosa* on *Brassica napus* roots was evaluated; MeOH 1.25% had no impact on parasitism (**Figure S6**). Ten pots per conditions were used for the DAPG experiment using a mixture containing natural soil artificially infested with approximatively 300 *P. ramosa* seeds per liter of soil. As described in the Experimental procedure section, plants were grown under controlled conditions with a 16h light and 8h dark photoperiod, at 25°C, with 50-70% relative humidity for 50 days. Treatments were applied twice during the experiment. The first application was performed when *B. napus* had between 2 to 4 leaves. The second application was done at 6 to 8 leaves. At harvest, broomrapes attached to root systems were collected, and counted. The horizontal lines indicate the interquartile range with the center representing the median. **(B)**: Proportion of broomrapes attached to the root of *B. napus* according to their developmental stage. The developmental stage of *P. ramosa* was estimated according to the developmental scale available in **Figure S4**. The number in the center of pie charts represents the mean of attached *P. ramosa* on *B. napus* roots and the size of pie charts is proportional to this number. Statistical differences are indicated with letters (ANOVA and Fisher’s LSD tests, p < 0.05).

**
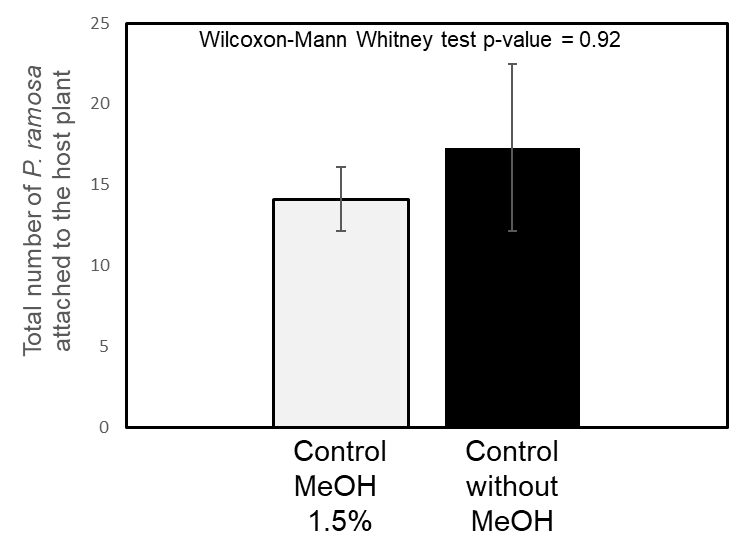
**

**Figure S6: Evaluation of the impact of MeOH 1.5% contained in buffer on the infection level by *P. ramosa* pv. oilseed rape on *Brassica napus* in greenhouse conditions.** The control conditions correspond to the application of 5 ml with 1 mM phosphate buffer pH 7.5 supplemented or not with 1.25% of methanol.


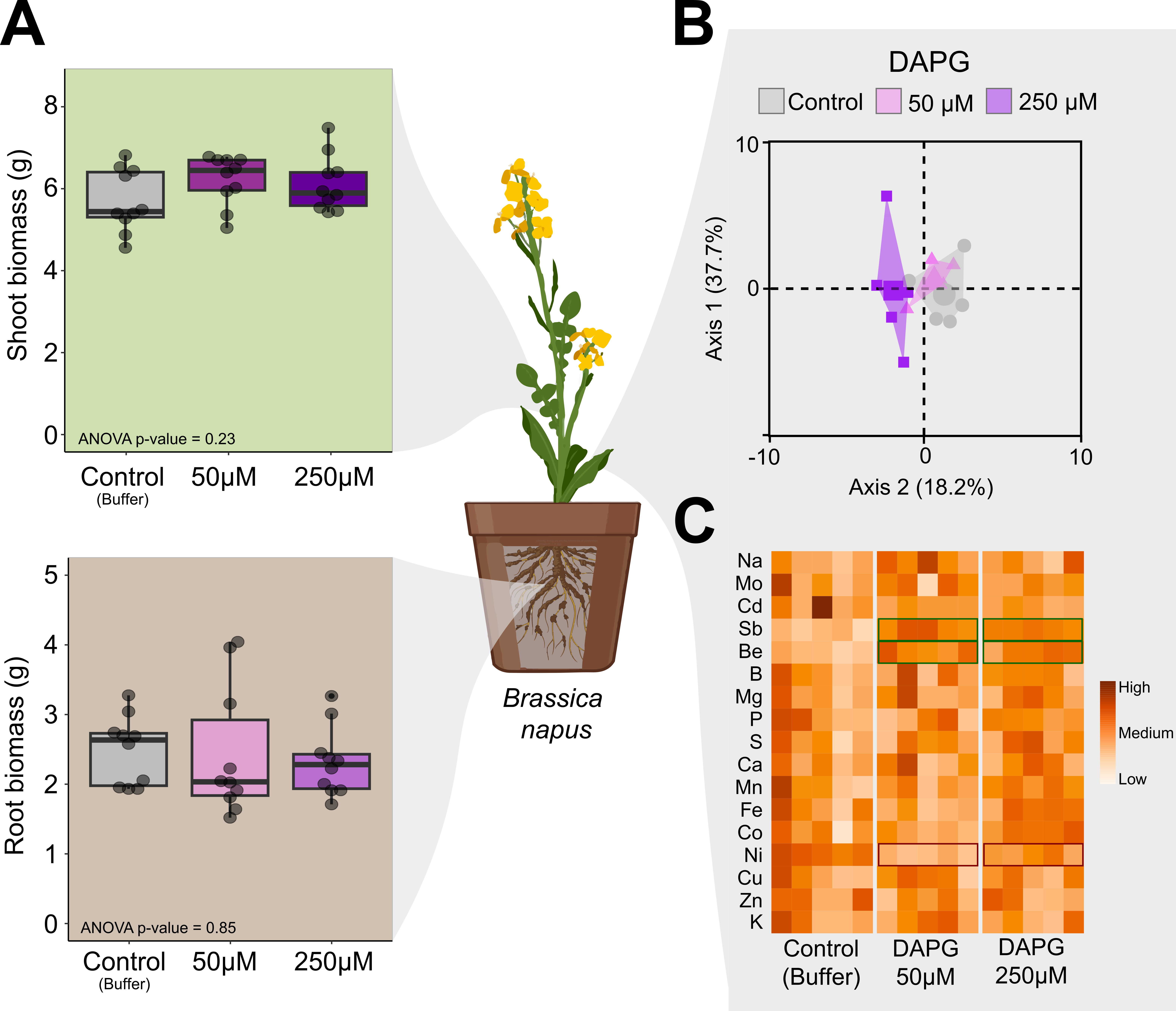


**Figure S7: Effect of the application of DAPG on the root and shoot biomasses and, on the ion profile of the shoot of *Brassica napus****.* **(A)** We measured for all the tested conditions the shoot and root biomasses of *Brassica napus* 50 days after sowing. For DAPG experiment, the control corresponds to the application of 5 ml with phosphate buffer supplemented with 1.25 % of methanol. The brown background of the boxplots corresponds to the root biomass data while the green background is associated with shoot biomass data. Statistical differences are indicated with letters (ANOVA and Fisher’s LSD tests, p < 0.05). The horizontal lines indicate the interquartile range with the center representing the median. **(B)**: Principal component analysis of the element composition of the shoot of *Brassica napus* according to the different treatments applied**. (C)**: Heatmap showing the elemental profile of the different conditions. Significant differences between treatments and control were obtained with Student’s t-test. When Student’s t-test assumptions were not met, a Welch t-test was performed. *p-value < 0.05, **p-value < 0.01. The detailed data are available in **Table S3.**

**Table S1:** List of the bacterial strains used in this study.

| **Strain names** | **Genotype or relevant characteristics**^1^ | **Reference or source** |
| --- | --- | --- |
| F113 | *Pseudomonas ogarae* F113 wild type | [1] |
| Δ*phlD* | Deletion mutant of *phlD*, impaired in the production of DAPG. | [2] |
| Δ*phlD Comp* | Δ*phlD* containing the low-copy plasmid pBBR1-MCS5:*phlD*, restoration of the DAPG production. Gm^R^ | [2] |
| Over *phlD* | F113 wild type containing the low-copy plasmid pBBR1-MCS5:*phlD*, enhancement of the DAPG production. Gm^R^ | This work |

^1^ Gm^R^, gentamicin resistance.

**Table S2:**Impact of the inoculation of F113 or Δ*phlD* on the elemental composition of the leaves of *B. napus*. (µg g^-1^ of dry biomass, except for Mg, P, S, Ca, Fe and K i.e., mg g^-1^ of dry biomass).

|  | Control | | F113 | | *ΔphlD* | |
| --- | --- | --- | --- | --- | --- | --- |
|  | **Mean** | *Average deviation* | **Mean** | *Average deviation* | **Mean** | *Average deviation* |
| **Shoot dry weight (g)** | 4.3 | *0.2* | 4.1 | *0.2* | 4.1 | *0.1* |
| **Number of attached *P. ramosa*** | 20.1 | *1.1* | 10.7 | *1.4* | 18 | *1.7* |
| **Na** | 3,923.89 | *374.92* | 5,545.03* | *700.82* | 4,610.88 | *832.7* |
| **K** | 125.74 | *12.90* | 132.62 | *15.49* | 120.73 | *10.11* |
| **Mo** | 78.99 | *9.72* | 60.55 | *17.08* | 66.32 | *11.92* |
| **Cd** | 1.14 | *0.19* | 0.84 | *0.19* | 1.02 | *0.11* |
| **Sb** | 0.10 | *0.03* | 0.15 | *0.057* | 0.06 | *0.013* |
| **Be** | 0.07 | *0.02* | 0.07 | *0.01* | 0.07 | *0.02* |
| **B** | 158.28 | *15.78* | 133.64 | *24.23* | 137.89 | *11.96* |
| **Mg** | 39.81 | *2.57* | 38.16 | *3.4* | 37.34 | *2.73* |
| **P** | 27.17 | *2.33* | 23.65 | *2.35* | 23.32* | *1.12* |
| **S** | 86.98 | *4.82* | 79.31 | *9.14* | 82.34 | *5.43* |
| **Ca** | 188.25 | *15.50* | 180.74 | *13.95* | 187.01 | *12.39* |
| **Mn** | 220.51 | *12.55* | 192.44* | *14.87* | 201.84 | *20.94* |
| **Fe** | 1.53 | *0.60* | 1.16 | *0.25* | 1.08 | *0.52* |
| **Co** | 1.03 | *0.29* | 0.8 | *0.15* | 0.86 | *0.36* |
| **Ni** | 3.00 | *0.49* | 3.06 | *0.96* | 3.17 | *0.74* |
| **Cu** | 30.29 | *1.94* | 28.1 | *2.37* | 28.57 | *2.57* |
| **Zn** | 192.14 | *50.90* | 146.92 | *21.29* | 165.45 | *10.59* |

Sodium (Na), Molybdenum (Mo), Cadmium (Cd), Antimony (Sb), Beryllium (Be), Bore (B), Magnesium (Mg), Phosphorus (P), Sulphur (S), Calcium (Ca), Manganese (Mn), Iron (Fe), Cobalt (Co), Nickel (Ni), Copper (Cu), Zinc (Zn), Potassium (K). n=5 samples of pooled of four plants. Significant differences between treatments and control were assessed using either t-tests or Welch t-test when Student’s t-test assumptions were not met. *, p<0.05; **, p<0.01. Red and green percentages correspond to significant negative and positive impact on ionome respectively.

**Table S3:**Impact of the application of two concentrations of DAPG on the elemental composition of the leaves of *B. napus*. (µg g^-1^ of dry biomass, except for Mg, P, S, Ca, Fe and K i.e., mg g^-1^ of dry biomass).

|  | Control | | DAPG 50 µM | | DAPG 250 µM | |
| --- | --- | --- | --- | --- | --- | --- |
|  | **Mean** | *Average deviation* | **Mean** | *Average deviation* | **Mean** | *Average deviation* |
| **Shoot dry weight (g)** | 5.3 | *0.18* | 6.15 | *0.19* | 6.1 | *0.21* |
| **Number of attached *P. ramosa*** | 15.4 | *2.4* | 11.4 | *2.1* | 15.3 | *1.9* |
| **Na** | 320.28 | *24.65* | 411.35^•^ | *62.92* | 353.63 | *59.27* |
| **K** | 66.87 | *10.01* | 68.43 | *7.12* | 63.46 | *6.44* |
| **Mo** | 48.67 | *4.88* | 49.49 | *4.16* | 47.78 | *1.45* |
| **Cd** | 0.84 | *0.43* | 0.57 | *0.02* | 0.54 | *0.05* |
| **Sb** | 0.03 | *0.01* | 0.12* | *0.05* | 0.10*** | *0.01* |
| **Be** | 0.052 | *0.002* | 0.063** | *0.004* | 0.063** | *0.003* |
| **B** | 94.23 | *9.74* | 97.37 | *13.99* | 94.95 | *5.81* |
| **Mg** | 18.06 | *1.74* | 18.71 | *2.04* | 18.96 | *1.93* |
| **P** | 16.92 | *2.52* | 15.91 | *2.06* | 16.55 | *0.57* |
| **S** | 24.24 | *2.2* | 24.54 | *1570.8* | 25.7 | *2.23* |
| **Ca** | 99.85 | *11.15* | 103.94 | *16.9* | 103.81 | *13.21* |
| **Mn** | 152.49 | *17.05* | 149.76 | *7.74* | 158.29 | *5.6* |
| **Fe** | 349.61 | *60.9* | 286.13 | *21.79* | 373.13 | *34.86* |
| **Co** | 0.45 | *0.07* | 0.42 | *0.03* | 0.52 | *0.03* |
| **Ni** | 1.79 | *0.47* | 0.34* | *0.07* | 0.76* | *0.27* |
| **Cu** | 12.44 | *1.56* | 12.96 | *1.25* | 12.51 | *1.01* |
| **Zn** | 84.42 | *8.94* | 77.3 | *6.73* | 79.39 | *9.34* |

Sodium (Na), Molybdenum (Mo), Cadmium (Cd), Antimony (Sb), Beryllium (Be), Bore (B), Magnesium (Mg), Phosphorus (P), Sulphur (S), Calcium (Ca), Manganese (Mn), Iron (Fe), Cobalt (Co), Nickel (Ni), Copper (Cu), Zinc (Zn), Potassium (K). n=5 samples of pooled of two plants. Significant differences between treatments and control were assessed using either t-tests or Welch t-test when Student’s t-test assumptions were not met. •, p<0.1 *, p<0.05; **, p<0.01. Red and green percentages correspond to significant negative and positive impact on ionome respectively.

**References**

1. Shanahan P, O’sullivan DJ, Simpson P, Glennon JD, O’gara F. Isolation of 2,4-diacetylphloroglucinol from a fluorescent pseudomonad and investigation of physiological parameters influencing its production. *Appl Environ Microbiol* 1992; **58**: 353–358.

2. Vacheron J, Desbrosses G, Renoud S, Padilla R, Walker V, Muller D, et al. Differential contribution of plant-beneficial functions from *Pseudomonas kilonensis* F113 to root system architecture alterations in *Arabidopsis thaliana* and *Zea mays*. *MPMI* 2018; **31**: 212–223.
